# Post-hoc analysis of adverse events during the EPOCH trial: reconsidering dosimetry

**DOI:** 10.1007/s00259-026-07948-6

**Published:** 2026-05-29

**Authors:** Marnix Lam, David Kersting, William Harris, Eveline Boucher, Etienne Garin, Kirk Fowers, Riad Salem

**Affiliations:** 1 Department of Radiology and Nuclear Medicine, University Medical Center Utrecht, P.O. Box 85500, Utrecht, 3508 GA The Netherlands; 2 Department of Nuclear Medicine, University Hospital Essen, Essen, Germany; 3 Division of Hematology and Oncology, University of Washington, Seattle, WA USA; 4 Boston Scientific Corporation, Marlborough, MA USA; 5 Department of Nuclear Medicine, Cancer Institute Eugene Marquis, Rennes, France; 6 Department of Radiology, Northwestern University, Chicago, IL USA

**Keywords:** Colorectal carcinoma, Radioembolization, Yttrium, Dosimetry, Liver

## Abstract

**Background:**

EPOCH demonstrated superior progression free survival using yttrium-90 [^90^Y] glass microspheres radioembolization plus chemotherapy versus chemotherapy in colorectal cancer patients with liver metastases who had progressed following first-line oxaliplatin or irinotecan-based chemotherapy. Exploratory post-hoc analyses and theoretical modeling assessed the potential impact of dosimetry on safety outcomes.

**Patients and methods:**

Liver-related treatment-emergent adverse event (TEAE) frequency and/or severity, and bilirubin increase were explored in participating patients. For hypothesis generation, a theoretical model was constructed to understand EPOCH dosimetry in the absence of multicompartment dosimetry data.

**Results:**

Liver-related TEAEs were reported in 93/186 (50%) patients, TEAEs ≥ grade 3 in 44/186 (24%) patients. A total of 26/186 patients (14%) experienced a bilirubin increase TEAE of any grade, in five of which assessed as related to glass [^90^Y]Y-radioembolization with a median fractional tumour involvement of 2.3% (range 1.4–4.1%). Under the theoretical assumption of a T/N ratio of 2–10, most patients in EPOCH (62% had a fractional tumour involvement < 10%) received a normal liver absorbed dose of at least 75 Gy. In this model, at < 4.1% fractional tumour involvement, the normal liver absorbed dose approximates 120 Gy at any T/N ratio.

**Conclusion:**

When treatment is same-day whole liver at 120 Gy average absorbed dose, the normal liver absorbed dose is dependent on fractional tumour involvement.

**Trial Registration:**

ClinicalTrials.gov NCT01483027.

## Introduction

In the EPOCH study, glass [^90^Y]Y-radioembolization (TheraSphere^®^, Boston Scientific) was added to second-line systemic therapy (i.e., irinotecan- or oxaliplatin-based chemotherapy) in colorectal cancer patients who progressed on first-line therapy [1]. Patients were randomly (1:1) assigned to radioembolization plus second-line systemic therapy in the treatment arm (215 patients of whom 187 received radioembolization) or control arm (i.e., standard of care second-line irinotecan- or oxaliplatin-based chemotherapy, 213 patients). The hazard ratio (HR) for PFS was 0.69 (95% CI, 0.54 to 0.88; 1-sided *P* = 0.0013), with a median PFS of 8.0 and 7.2 months, respectively. Although positive, the study was criticized for the lack of overall survival (OS) and/or quality of life benefit, and for the observed toxicity and risk of hepatic decompensation [2–4].

Additional exploratory analyses of EPOCH identified subgroups with enhanced response rates, (hepatic) PFS, reduced toxicity by event rate/100 patients’ years and a significant improvement in ‘time-to-deterioration-of-quality-of-life’ in the combination-arm in comparison to systemic therapy alone [5]. Identifiers of these subgroups (i.e., ECOG 0, CEA < 35 ng/mL, KRAS wildtype) may be used for improved patient selection.

Furthermore, editorials suggested that advances in dosimetry-based treatment planning are critical for further improvement and should be studied in more detail [5]. The aim of this post-hoc analysis of EPOCH study data therefore was: (1) to further evaluate observed liver-related toxicity; (2) to perform theoretical post-hoc dosimetry modeling for EPOCH.

## Methods

Eligibility criteria included age ≥ 18 years, unresectable unilobar or bilobar mCRC, able to receive second-line irinotecan- or oxaliplatin-based chemotherapy, measurable disease by RECIST 1.1, performance status 0 or 1, bilirubin ≤ 1.2 times upper limit normal, and albumin ≥ 3.0 g/dL. Key exclusion criteria were prior arterial therapy or radiotherapy to the liver, clinically evident ascites, unresolved toxicities from first-line therapy, confirmed extrahepatic metastases, or contraindications to angiography.

Reported liver-related ‘treatment-emergent adverse events’ (TEAE) included liver enzymes, ascites, oedema, bilirubin, albumin, hepatic failure, encephalopathy, bile duct obstruction, cholecystitis, cholangitis, hepatic/portal vein thrombosis, liver abscess and INR (CTCAE v3.0 data available only). In the present analysis, bilirubin increase was analyzed separately [1].

**Fig. 1:**
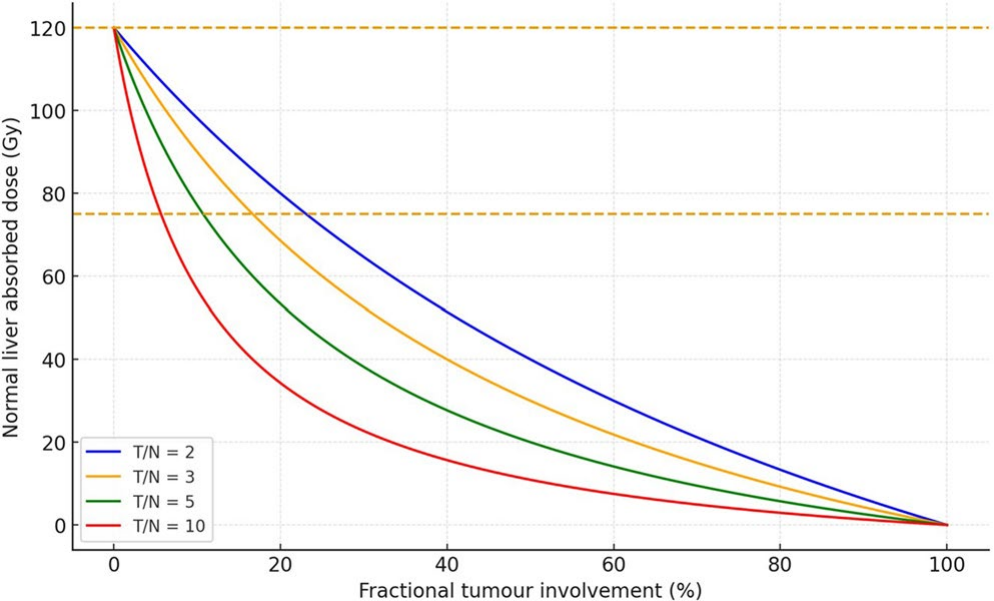
Normal liver absorbed dose in Gy as a function of fractional tumour involvement in patients treated in a single session whole liver approach at 120 Gy average absorbed dose. The whole liver weight is 1.5 kg, the tumour-to-non-tumour (T/N) ratio ranges from 2–10, the lung shunt is 0%. Dotted yellow lines at 75 Gy (currently recommended maximum normal liver absorbed dose) and 120 Gy. Note: at higher T/N ratios the normal liver absorbed dose decreases as a function of fractional tumour involvement due to preferential flow to the tumour

Activity was prescribed using single-compartment 120 Gy average absorbed dose to the perfused volume. Since post-treatment imaging was not performed and pretreatment technetium-99m [^99m^Tc]Tc-MAA SPECT/CT was not collected, tumour and normal liver absorbed doses could not be calculated. To better understand dosimetry in EPOCH in the absence of multicompartment data, a theoretical model was used. Assuming no lung shunt and a specific tumour-to-non-tumour (T/N) ratio, both the tumour absorbed dose and the normal liver absorbed dose will only depend on the fractional tumour involvement of the liver (Fig. 1): Normal liver absorbed dose in Gy = 120 Gy / (1 + fractional tumour involvement (T/N ratio – 1)). If the T/N ratio equals 1, the normal liver absorbed dose equals the average absorbed dose (i.e., 120 Gy in EPOCH), it decreases with increasing fractional tumour involvement and T/N ratio.

Hypotheses were generated by combining observed toxicity and theoretical modeling.

## Results

Out of 187 treated, 186 patients were evaluable. Most patients had bilobar disease (82%), received whole liver treatment in one session (64%), had > 5 tumours (68%), and limited fractional tumour involvement < 10% (62%). Liver-related TEAEs were reported in 93/186 (50%) patients, TEAEs **≥** grade 3 in 44/186 (24%) patients. Fatal liver related death occurred in 6/186 (3%) patients, 3/186 classified as REILD, in the other three it was related to progressive disease and/or systemic treatment (Table 1). A significant relationship was found between number of tumours and TEAEs, but not for fractional tumour involvement.

**Table 1:** Liver-related treatment emergent adverse events and associated disease characteristics in glass [^90^Y]Y-radioembolization patients (*N* = 186)

Number of lesions	Fractional tumour involvement (%)	Patients (*N*)	Liver related TEAEs *N* (*n*) (%) ^#^	Liver related grade ≥ 3 TEAEs *N* (*n*) (%)	Fatal liver related events (*N*)
< 3	< 10	19	8 (22) (42)	2 (2) (11)	
	≥ 10 to < 25	1	0	0	
	≥ 25	1	0	0	
3–5	< 10	32	11 (38) (34)	9 (15) (28)	1
	≥ 10 to < 25	6	2 (3) (33)	1 (1) (17)	
	≥ 25	1	0	0	
6–10	< 10	35	23 (83) (66)	11 (23) (31)	
	≥ 10 to < 25	11	4 (6) (36)	1 (1) (9)	
	≥ 25	4	2 (5) (50)	0	
> 10	< 10	29	14 (47) (48)	7 (12) (24)	2
	≥ 10 to < 25	32	20 (45) (63)	9 (15) (28)	1
	≥ 25	15	9 (22) (60)	4 (6) (27)	2

*N *Number of patients, *n *Number of events, *TEAE *Treatment Emergent Adverse Events

# A significant association was found with number of lesions (Fisher’s Exact *P* = 0.04). No other significant associations were found in the presented data

**Fig. 2:**
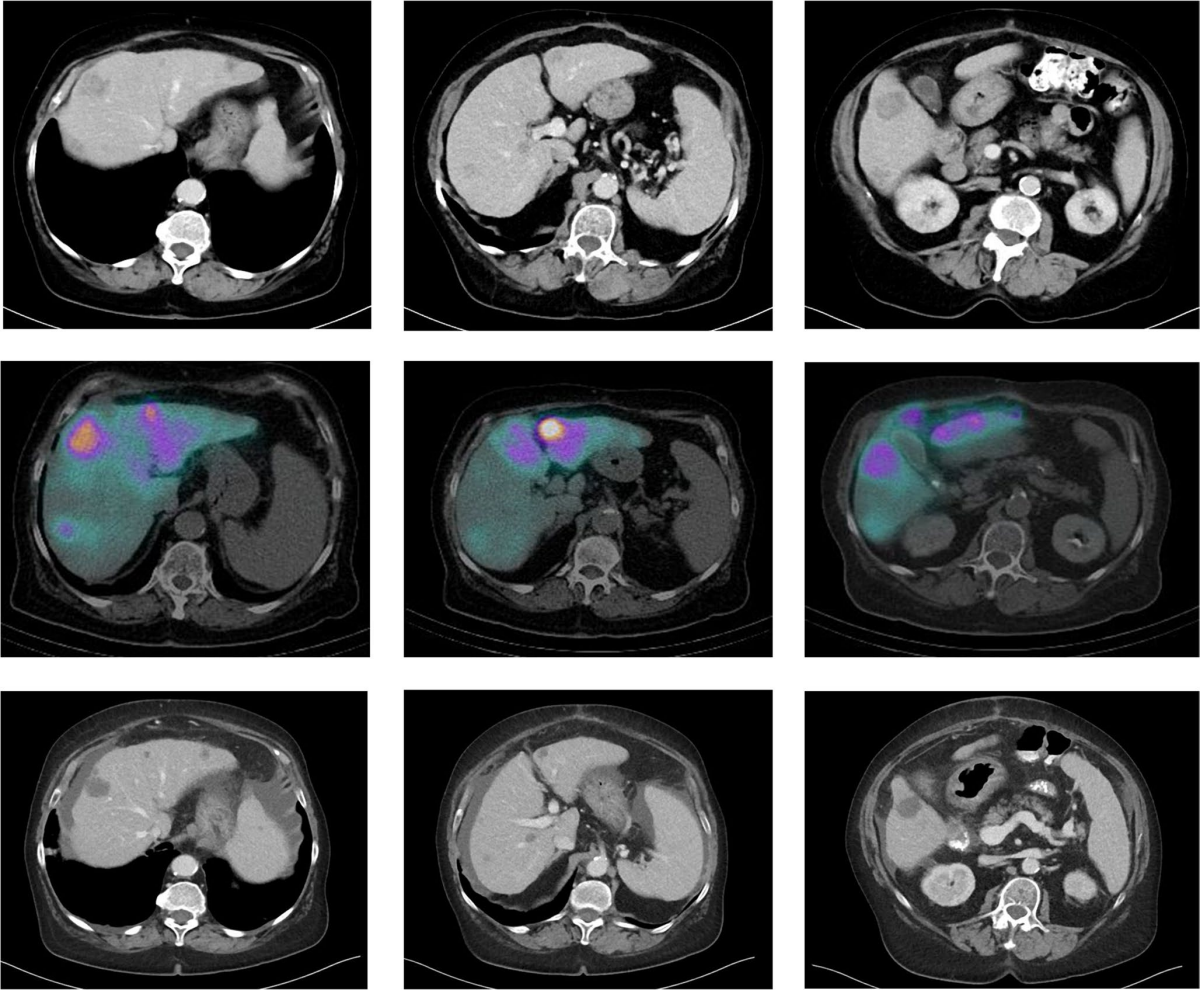
A 68-year-old female patient with adenocarcinoma of the coecum (in situ) and multiple small liver metastases (maximum diameter 3 cm, fractional tumour involvement < 5%; upper row) received FOLFOX plus bevacizumab and same-day whole liver radioembolization (2.17 GBq right hepatic artery; 1.62 GBq left hepatic artery; middle row). She experienced progressive ascites and increasing bilirubin levels 2.5 months after treatment, while lesions were stable in size

A total of 26/186 patients (14%) experienced a bilirubin increase AE of any grade (eight grade 1, 10 grade 2, five grade 3, two grade 4, one grade 5). The bilirubin increase was related to glass [^90^Y]Y-radioembolization in 5/186 patients (2.7%; three grade 2 and two grade 3 toxicity; Table 2) and unrelated in 21 patients. Reported reasons for unrelatedness included chemotherapy-related toxicity, disease progression and concurrent disease. The median fractional tumour involvement in 26 patients with any bilirubin increase was 5% (range 0.4–50.6%) and 2.3% (range 1.4–4.1%) in the five patients with glass [^90^Y]Y-radioembolization related toxicity, an incidence of 5/77 (6%) at fractional tumour involvement < 4.1%. In these five patients with related toxicity, the median interval between glass [^90^Y]Y-radioembolization treatment and incidence of bilirubin increase was 3.2 months (range 2.1–5.3 months). In 2/5 patients, hyperbilirubinemia was considered REILD, i.e., accompanied by grade 2–3 ascites and grade 2 hypoalbuminemia. Best response was partial response in four and stable disease in one patient (Fig. 2). Median CEA decrease was 60% (range 21.6–93.2% decrease). Median overall survival from glass [^90^Y]Y-radioembolization was 13.4 months (range 6.1–23.7 months).

**Table 2:** Characteristics of five patients with treatment related bilirubin increase

Median (range) age in years	57 (46–68)
KRAS mutant / wildtype	4 / 1 patient(s)
ECOG status 0 / 1	5 / - patients
First-line systemic therapy oxaliplatin-based / irinotecan-based	3 / 2 patients
Median (range) hepatic tumor load in %	2.3 (1.4–4.1)
Number of hepatic tumors	Patients
< 3	1
3–5	2
6–10	1
> 10	1
Treatment fraction	100% in all
Median (range) average absorbed dose in Gy	121.3 (109.4-130.9)
Treatment week	Patients
Week 1	2
Week 2	1
Mixed week 1 / week 2	2
Median (range) baseline laboratory values	
CEA in ng/mL	13.3 (3.7–1314)
Bilirubin in umol/L	9.8 (6-18.8)
Albumin in g/dL	4.2 (3.4–4.6)
Alanine aminotransferase in IU/L	26 (10–121)
Aspartate aminotransferase in IU/L	22 (19–136)
Alkaline phosphatase in IU/L	132 (92–174)

Figure 1 shows how fractional tumour involvement affects normal liver absorbed dose if patients receive 120 Gy average absorbed dose (as done in EPOCH). Under the theoretical assumption of a T/N ratio of 2–10, most patients in EPOCH (62% had a fractional tumour involvement < 10%) received a normal liver absorbed dose between 75 Gy and 100 Gy. In this model, at < 4.1% fractional tumour involvement, the normal liver absorbed dose approaches 120 Gy at any T/N ratio.

## Discussion

The incidence of toxicity related to glass [^90^Y]Y-radioembolization was acceptable, as previously reported [6]. Incidence of bilirubin increase related to glass [^90^Y]Y-radioembolization treatment was low (2.7%) and comparable to rates noted for glass [^90^Y]Y-radioembolization alone. While incidence was acceptably low, all treatment-related bilirubin increase occurred at very low fractional tumour involvement (< 5%). These five patients received same-day whole liver treatment using per-protocol 120 Gy ± 10% average absorbed dose, resulting in normal liver absorbed doses approaching 120 Gy (Fig. 1), above the current recommended 75 Gy threshold [7]. However, given the low incidence of toxicity in EPOCH and other studies [8, 9], the preliminary safety threshold of 75 Gy (whole liver) normal liver absorbed dose for glass [^90^Y]Y-radioembolization may be too conservative. Dosimetry-based treatment planning (alongside appropriate patient selection) further increases efficacy [8], but may also prevent toxicity, especially when appropriate tumour dose alongside particle density are considered [10].

Bilirubin increase may be regarded as a safety proxy for the occurrence of unacceptable hepatotoxicity [11]. The definition of liver-related toxicity in EPOCH was rather broad, which probably diluted dose-related effects, caused by concomitant treatments, illnesses and progressive disease. This might have been the reason why the relationship between TEAE and higher normal liver absorbed doses (with fractional tumour involvement as a surrogate) seemed to be lacking (Table 1).

Limitations of this post-hoc analysis include the lack of multicompartment dosimetry data. Although theoretical modeling was used, the assumptions made were reasonable, since most patients received whole liver treatment, all at 120 Gy average absorbed dose and a broad range was used for T/N. Future studies should nonetheless include multicompartment dosimetry treatment planning based on pretreatment [^99m^Tc]Tc-MAA SPECT/CT and post-treatment evaluation as part of their treatment protocol to identify a normal tissue absorbed dose limit, particularly in patients undergoing whole-liver treatment [12].

## Conclusion

EPOCH reported a low incidence of [^90^Y]Y-radioembolization toxicity. However, when using average absorbed doses as part of single-compartment dosimetry, one should take fractional tumour involvement and its consequence for normal liver absorbed dose into consideration.

## Data Availability

Data sharing requests can be made at: https://www.bostonscientific.com/en-US/data-sharing-requests/data-sharingrequest-submission-form.html.
